# The Strategies Used to Balance Health and Work Across the Solid Organ Transplantation Trajectory

**DOI:** 10.1177/15269248241239245

**Published:** 2024-04-05

**Authors:** Keira Gaudet, Marc Corbiere, Tianyan Chen, Heloise Cardinal, Marie Achille

**Affiliations:** 1 Department of Psychology, 5622 Universite de Montreal, Montreal, Quebec, Canada; 2 Department of Education, Career Counselling, Universite du Quebec a Montreal (UQAM), Montreal, Quebec, Canada; 3 Department of Medicine, Faculty of Medicine and Health Sciences, McGill University, Montreal, Quebec, Canada; 4 Division of Gastroenterology and Hepatology, McGill University Health Center (MUHC), Montreal, Quebec, Canada; 5 Department of Medicine, 5622 Universite de Montreal, Montreal, Quebec, Canada; 6 Department of Nephrology, Centre hospitalier de l'Universite de Montreal (CHUM), Montreal, Quebec, Canada

**Keywords:** solid organ transplantation, job retention, self-management, coping strategies, help-seeking, workplace accommodations

## Abstract

**Introduction:** Workers who undergo solid organ transplantation report frequent conflicts between the unpredictable demands of their health condition and the rigid requirements of their employer and of health services. The present study aimed to describe the self-management strategies adopted by workers while staying at work before transplantation and during sustainable return-to-work posttransplantation. **Methods:** Fifteen employed kidney, liver, and lung transplant recipients were recruited from 2 large urban university health centers in Montreal, Canada. Three focus groups were held, and thematic analysis was performed. **Findings:** Seven strategies were identified: responding promptly and consistently to fatigue-related needs, planning ahead with immediate supervisors while remaining strategic about when to disclose transplantation, requesting work accommodations, requesting flexibility in healthcare provision, consulting physicians about work-related issues, informing co-workers about work limitations and immunosuppression and asking not to be treated differently in the workplace. **Conclusion:** Access to work accommodations, support from physicians and flexibility in treatment and appointment schedules supported workers’ ability to manage their health while staying at work before and after undergoing solid organ transplantation. In light of findings, it may be useful for healthcare professionals to address workers’ concerns about work limitations and work accommodation implementation, especially when the illness-management burden increases before transplantation and during posttransplantation sick leave. Future studies could describe the strategies used by other important stakeholders when attempting to provide support to workers.

## Introduction

Return-to-work (RTW) is considered a marker for successful posttransplantation rehabilitation by both healthcare professionals and solid organ transplant recipients.^1^ RTW after transplantation has been linked to lower depressive symptomatology and associated with lower risks of organ rejection and mortality.^2,3^ However, findings derived from US registry data have indicated that 38% of individuals become unemployed after the onset of an end-stage kidney disease.^4^ Rates of job loss were estimated to be equivalent or higher for end-stage liver, lung, and heart diseases.^5^ After transplantation, most unemployed individuals do not reintegrate into the workforce.^3,6^

Transplantation and end-stage organ diseases (ESODs) involve significant burdens of daily illness-management, including fatigue-related modifications in daily activities, frequent hospital visits, and navigating infection risks.^7,8^ Workers pre and posttransplantation have reported frequent conflicts in their daily lives between the unpredictable demands of their health condition and the rigid requirements of their employer and of health services.^9^

A key focus in work disability prevention involves the promotion of job retention among employed individuals by harnessing their strengths and resources.^10^ Self-management strategies encompass individuals’ efforts to address the impacts of chronic disease on their physical and mental health, including their ability to participate in work and family life.^11^ Using self-management strategies may increase workers’ perceived sense of self-efficacy in dealing with obstacles to employment maintenance.^12^ As they perceived their work environment and health situation as difficult to change, workers have expressed preferring to use strategies within their control.^13^ Fostering a sense of control and self-efficacy by using self-management strategies may be useful for workers who undergo transplantation and who face unpredictable prognoses and frequent health changes.

Prior qualitative studies have identified some self-management strategies employed by workers with an ESOD or undergoing organ transplantation. These strategies involved explaining symptoms to team members and requesting work accommodations (workload reduction, schedule flexibility).^9,14–16^ The reviewed studies focused on broad experiences of work maintenance^14,15^ or on specific sub-topics, that is, logistical challenges and social support.^9,16^ Because self-management strategies were not the main research topic, it is unclear how workers approached acquiring support from immediate supervisors, co-workers, and healthcare professionals. Findings from a scoping review on stakeholders’ roles and actions in the RTW process suggested that effective self-management in workers entailed proactively seeking support from managers, rehabilitation professionals, and insurers.^10^ Moreover, most of the studies that touched on the self-management strategies used by workers who underwent transplantation focused exclusively on either the pre or posttransplantation periods.^9,14–16^ Gaining knowledge about the strategies used by the same workers as they navigate the transplantation trajectory might help identify key moments when support from healthcare professionals and workplace stakeholders might be particularly impactful.

To understand how workers attempt to balance health and work demands as they navigate the transplantation trajectory, the present study aimed to describe the self-management strategies used by workers while staying at work before solid organ transplantation and during sustainable RTW after transplantation.

## Methods

### Design

An inductive descriptive qualitative research design, specifically the qualitative description approach, was employed.^17^ This approach focuses on straightforward descriptions of participant experiences by closely adhering to their discourse, in order to generate practical insights that are relevant to healthcare practices. The study was approved by the institutional review boards of both hospitals where it took place (multi-site project ID: MP-02-2021-9014). Informed consent was obtained from all participants.

### Sampling

Kidney, liver, and lung transplant recipients were recruited. Heart transplant recipients were excluded from the study since heart transplantation is managed outside of the scope of partner university health centers. Participants were recruited between January and June 2021 from 2 large university-affiliated hospitals in Montreal, Canada. Physicians met with follow-up nurses from each hospital to identify individuals who were likely to be employed and who had undergone transplantation in the preceding 2 years. A member of the hospital staff called each patient to ask whether they agreed to be contacted for the study. The first author then contacted patients to verify their eligibility.

Focus groups were held to foster participants’ disposition to reflect on their experiences and perspectives through interactions with other participants of diverse work and personal backgrounds.^18^ Three groups of 5 to 7 participants were conducted. A purposeful sampling strategy was used to obtain a diverse sample of transplant recipients meeting both general and specific criteria. The general inclusion criteria were: (*a*) Being 18 to 65 years old, (*b*) Being able to understand and speak French, (*c*) Having a functioning graft, (*d*) Having a job with the same employer then at the time of transplantation when the focus groups took place, (*e*) Having been back at work for at least 3 months and, at most, 2 years, and (*f*) Having perceived at least moderate levels of challenge to work participation while staying at work pretransplantation and/or during RTW posttransplantation. For this last criterion, used to facilitate the examination of coping behaviors,^11^ participants had to answer at least moderately to one or both of these questions: “How difficult or challenging was [staying at work before transplantation] / [returning to work after transplantation]? Not at all, slightly, moderately, very much or extremely.” For the duration of RTW (criterion *e*), 3 months is an accepted threshold for sustainable RTW in rehabilitation research.^19^ Two years was deemed an appropriate maximum time period for participants to be able to recollect pretransplant experiences with accuracy.^20^

Specific sampling criteria were: (*a*) At least 1 liver or lung transplant recipient per focus group, and (*b*) At least 4 individuals who shared a characteristic that constituted a risk factor for work disability (one characteristic per focus group): identifying as a woman, having been on sick leave for at least 6 months, or having a physically demanding job.^19,21^ Eligibility and sampling criteria are summarized in **Table 1**. The exclusion criteria were: (*a*) Having a cognitive impairment (self-reported), and (*b*) Planning to retire within the next 6 months.

**Table 1.** Participant Characteristics.

Characteristic (N = 15)	N (%)
**Age groups**	
Younger adults (32-44 y/o)	5 (33)
Middle-aged adults (45-54 y/o)	7 (47)
Older adults (55-61 y/o)	3 (20)
**Gender, women**	7 (47)
**Types of transplants**	
Kidney	11 (73)
Liver	2 (13)
Lung	2 (13)
**Types of dialyses (N = 11)**	
In-clinic	6 (55)
At home	4 (36)
No dialysis	1 (9)
**Job roles**	
Management	4 (26)
Professional (university degree required)	3 (20)
Technical (trade school or pre-university)	5 (33)
Intermediate (high school or job-specific training)	3 (20)
**Work duration before transplantation (symptom onset** ^a^ **to the start of sick leave)**	
3-11.9 months	4 (27)
1-1.9 years	5 (33)
2-4 years	6 (40)
**Sick leave duration**	
1-5.9 months	6 (40)
6-11.9 months	4 (27)
1-3.5 years	5 (33)
**Duration of return-to-work after transplantation**	
5-5.9 months	3 (20)
6-11.9 months	6 (40)
12-22 months	6 (40)
**Living situation**	
Partner and kids	7 (47)
Partner only	3 (20)
Alone	5 (33)

^a^ Symptom onset: when symptoms became bothersome on a daily basis according to self-reported data collected before the focus groups took place.

### Information Collection

Focus groups were conducted remotely using the Zoom video conferencing platform. Before the focus groups, the first author conducted individual phone interviews with each participant to collect socio-demographic data and to ask open-ended questions about pre and posttransplantation obstacles to work maintenance and self-management strategies.

For each focus group, 3 to 4 distinct set of strategies were selected for discussion. A semi-structured interview guide was used, which had been piloted with a test group. The main interview question was: “What strategies did you use to overcome the obstacles that you perceived to [staying at work pretransplantation] / [returning to work posttransplantation]? A strategy is any means by which you attempted to support your ability to keep working or your mental or physical health while staying at work.” Each focus group lasted approximately 120 minutes and was moderated by the first author. A co-moderator helped manage time, asked clarification questions, and met with the moderator at the end of each focus group to discuss salient results. Participants were encouraged to contribute additional insights through prompts such as invitations for comments. Moderators addressed participants who had provided responses on the topic discussed during phone interviews but remained silent during the discussion. Inquiries into shared experiences and diverse viewpoints were also made.

### Data Analysis

Thematic analysis was performed.^22^ The data involved transcribed interviews and detailed notes from the phone interviews. Rigor was assessed using the criteria recommended by Patton.^18^ To enhance the reliability of the coding process, 2 focus groups and their respective participants’ phone interview notes were independently coded and compared. Divergences were discussed, and problematic excerpts were re-coded until agreement was achieved. The first author coded the third focus group and its participants’ phone interview notes using the resulting code list. Similar codes were then grouped together to create themes. The initial set of themes was reviewed and modified in collaboration with the senior author, a clinician, and researcher specializing in the psychology of transplantation, to enhance credibility. Codes were re-read, and ambiguous or conflicting codes were subjected to further analysis. Transcribed interviews were also re-read to verify that the themes adequately represented participants’ experiences. Particular attention was given to representing the experiences of individuals with the targetted work disability risk factors (being a woman, having been on sick leave for at last 6 months and having a physically demanding job). This procedure aimed to foster the application of findings beyond the specific study setting (transferability).

## Findings

### Participant Characteristics

From the 58 transplant recipients who were contacted, 32 did not meet the general eligibility criteria (including 5 who perceived little to no challenge to their work maintenance), 7 refused to participate, and 4 had scheduling conflicts. Fifteen participants comprised the sample. Main participant characteristics are displayed in **Table 1**.

### Self-Management Strategies Described by Participants

Seven themes were identified, each comprising strategies that encapsulated narratives expressed by one or more participants, pertaining to either one or both time periods of interest: staying at work before transplantation and sustainable RTW after transplantation. The self-management strategies are summarized in **Table 2** and described in detail below.

**Table 2.** Self-Management Strategies Used During Sustained Work Participation Before and After Solid Organ Transplantation.

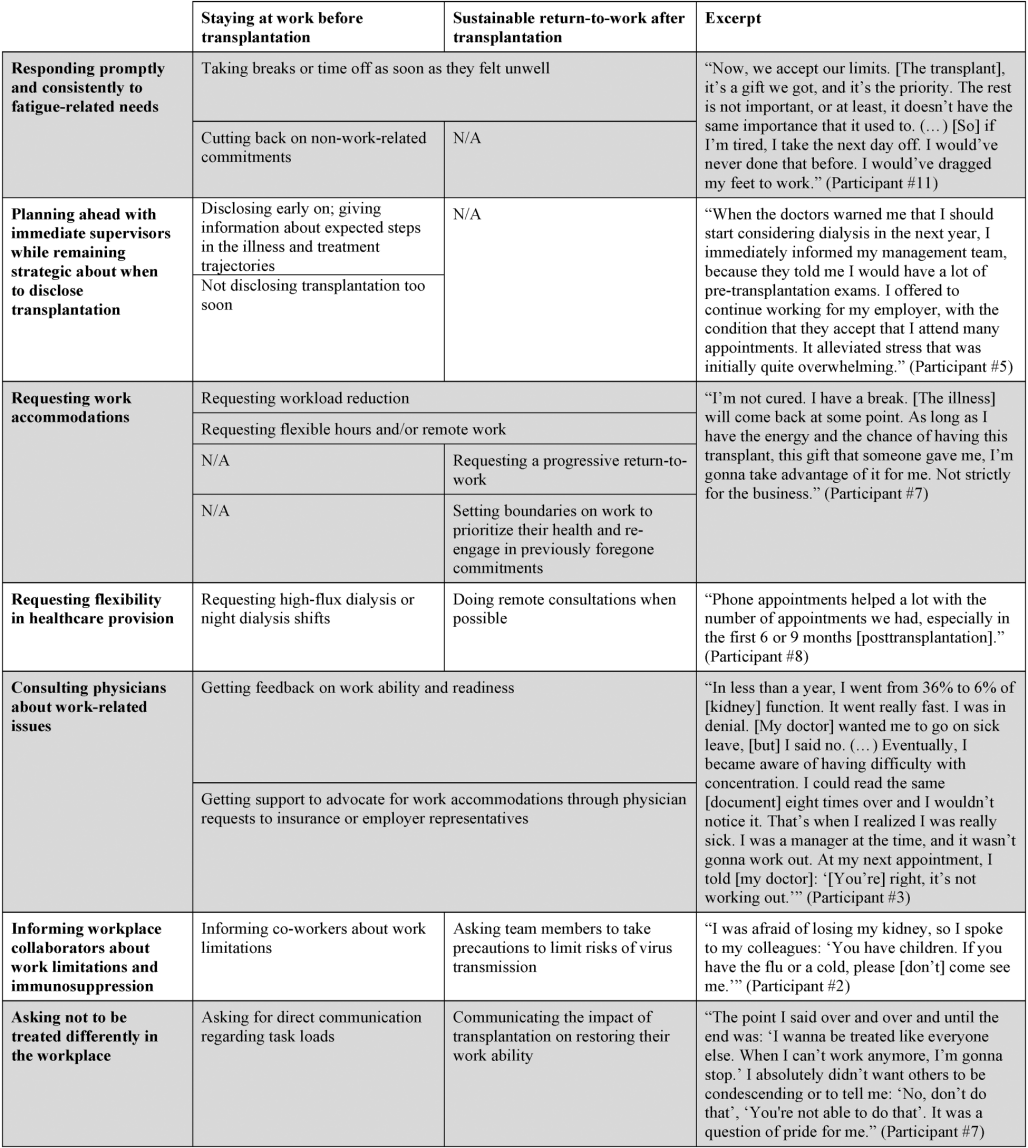

N/A, Not Applicable.

### Responding Promptly and Consistently to Fatigue-Related Needs

**Staying at work before transplantation—strategies used.** Almost all participants described experiencing high levels of fatigue pretransplantation. Many of them reported that taking breaks or time off as soon as they felt unwell helped them preserve enough energy to finish their day's or their week's work. This strategy also helped a few participants address pain, nausea, concentration issues, and difficulty breathing. A participant with a physically demanding job noted that this strategy helped prevent further work disability: “[My boss] preferred having me at home, resting for a day, than having me be absent for several days after that.” (Participant #14) Some participants also described cutting back on non-work-related commitments by limiting their involvement in hobbies, social activities, and family life. They explained that most of their already limited energy and time was mobilized by work, medical appointments, and dialysis.

**Sustainable RTW after transplantation—strategies used.** Most participants reported some fatigue in the first few months to a year following transplantation and kept taking breaks or time off when needed until fatigue levels eventually diminished.

### Planning Ahead With Immediate Supervisors While Remaining Strategic About When to Disclose Transplantation

**Staying at work before transplantation—strategies used.** When the ESOD was diagnosed, many participants were given the option to go on sick leave by their physician. Disclosing the ESOD to their immediate supervisor helped them determine if they could access work accommodations, which would allow them to keep working. Most participants did so early in the disease's progression. They underlined the importance of giving information about expected steps in their illness and treatment trajectory by explaining how their work ability might be impacted as the disease progressed. Two participants reported not disclosing the ESOD or transplantation too soon out of fear of losing either their job or access to a promotion: “I went step by step, to avoid scaring them right away.” (Participant #3)

### Requesting Work Accommodations

**Staying at work before transplantation—strategies used.** Dialysis, for those with end-stage kidney disease, and medical appointments often conflicted with participants’ work schedules. Work accommodations aimed to address these conflicts and facilitate fatigue-management. Participants asked for a reduced task load, for example, physical tasks or work hours. Some participants made smaller task adjustments, setting limits on requests that were outside of their role or sticking to familiar tasks to address concentration issues. When possible, participants adopted flexible hours and favored remote work. The uptake of these practices occurred at various points relative to the COVID-19 pandemic and the peak of social isolation measures. Notably, some participants highlighted that the pandemic culture positively influenced the acceptance of remote work. Some of those who had fixed shifts made themselves available to take work calls during dialysis. Some participants reported that these strategies, combined with taking time off to rest, helped them “stay in shape for the [transplant] surgery” (Participant #12) by limiting risks of injury or exhaustion.

Even though they implemented work accommodations, some participants felt that staying at work before transplantation impacted their general health and well-being. Those with young children felt that they did not spend enough time with their family, and most of those in physically demanding jobs described feeling that they had put their health at risk by pushing their body beyond its limits.

**Sustainable RTW after transplantation—strategies used.** Participants described maintaining flexible hours and remote work in the first few months after RTW, since immunosuppression levels were high and medical appointments were frequent. Most participants also implemented a progressive RTW to fend off posttransplantation fatigue by resting between shifts and taking breaks when needed. This strategy was seen as especially helpful by those who had been on long-term sick leave.

Many participants explained having learned from their pretransplantation experiences of having pushed themselves beyond their comfort levels. After transplantation, they described wanting to enjoy their lives while the transplant allowed them to do so. To care for the transplanted organ, these participants set boundaries on work to preserve their health and re-engage in previously foregone leisure activities and family commitments. Some, especially those with physically demanding jobs, made sure that they got access to sustainable workload reduction targeted at preventing injury or exhaustion. Others made clear that they were not to do overtime or take on extra tasks.

### Requesting Flexibility in Healthcare Provision

**Staying at work before transplantation—strategies used.** Getting access to flexibility in the provision of healthcare helped some participants address the conflicting demands of illness and work. A few, who had fixed work shifts, reported requesting high-flux dialysis or night dialysis shifts. One participant explained his work situation to the clinic personnel who, as an exception, facilitated his access to the coveted night dialysis shifts.

**Sustainable RTW after transplantation—strategies used.** Remote consultations were implemented by health centers as a response to the COVID-19 pandemic. Many participants agreed that this measure ultimately facilitated their RTW: “I live far from the hospital. [I had to] spend hours just to see my doctor. Now, he calls me, I talk to him for 15 minutes, and he gives me the exact same information than when I see him in person.” (Participant #10)

### Consulting Physicians About Work-Related Issues

**Staying at work before transplantation—strategies used.** When their health started worsening, some participants described having difficulty accepting their work limitations. A few reported that discussing their limitations with their nephrologist, hepatologist, or pulmonologist helped them come to terms with having to make significant changes if they wanted to keep working: “[My doctor] said: ‘You think you have the strength [to RTW], but you don’t. We all think we’re superman.’ And she was right. I didn’t have the strength.” (Participant #15) When facing employer or insurance-related barriers, a few participants got support from their physician, who helped identify work accommodations and forwarded written requests to insurance or employer representatives.

**Sustainable RTW after transplantation—strategies used.** Participants described that collaborating with their posttransplantation nephrologist, hepatologist, or pulmonologist was especially helpful when they were on sick leave during their recovery. Specialized physicians held similar roles, helping participants address work limitations and implement work accommodations.

### Informing Co-Workers About Work Limitations and Immunosuppression

**Staying at work before transplantation—strategies used.** A few participants reported being the target of comments regarding their work absences or reduced productivity, which made them feel judged and misunderstood: “It didn’t look like [I] was sick. But it's inside, you can’t see it! It's as if I was missing a leg, and I kept running anyway.” (Participant #12) Some participants reported that informing their co-workers about ESOD-related changes in work ability helped prevent such comments: “[They knew that] it wasn’t because I didn’t wanna work, it was because I couldn’t.” (Participant #4)

**Sustainable RTW after transplantation—strategies used.** When they returned to work, some participants reported feeling scared of catching infections due to immunosuppressive anti-rejection medication. Those fears sometimes involved anxiety about graft loss due to virus-induced organ rejection. These participants described asking co-workers and clients to take precautions, that is, to keep a distance, to use hand sanitizer and to stay away if they were sick or had been in contact with someone who was sick. Helpful approaches included addressing this issue with everyone at work, doing it as soon as they got back, being direct as opposed to trying to soften the message through humor and explaining why these precautions were important.

### Asking Not to be Treated Differently in the Workplace

**Staying at work before transplantation—strategies used.** Even though participants considered that illness disclosure at work was necessary to get support, some struggled with being seen as sick. To preserve their “pride,” these participants reported asking for direct communication regarding task load. They invited team members to address them directly and to let them adjust their own task load instead of assuming the extent of their work limitations.

**Sustainable RTW after transplantation—strategies used.** Once they returned to work, a few participants reported that their co-workers insisted on helping them, even though their work ability was mostly recovered: “It makes you go back, and you’re trying to move forward. (…) I mean, we’re still gonna be on pills. Except that it's not a disease to be a transplant recipient.” (Participant #14) Communicating the impact of transplantation on restoring their work ability helped these participants move away from the sick role while facilitating teamwork. Follow-up nurses and other healthcare professionals “gave [some participants] the language to communicate” (Participant #5) their new post-transplantation realities effectively.

## Discussion

The present study aimed to identify the self-management strategies used by workers when staying at work before solid organ transplantation and during sustainable RTW posttransplantation. As an overarching finding, workers tended to implement self-management strategies at 2 key moments in their transplantation trajectory: (*a*) Around the onset of the ESOD, and (*b*) During posttransplantation sick leave/recovery. These periods were marked by increased fatigue and hospital visits, which seemingly prompted support-seeking behaviors.

A first set of strategies involved workers proactively communicating their situation and needs to relevant stakeholders. As ESOD-related symptoms, treatments, and follow-ups became burdensome pretransplantation, all participants partially or fully disclosed their health condition to their immediate supervisor. Timely access to work accommodations potentially contributed to participant job retention, which aligns with previous findings. A recent systematic review highlighted strong evidence linking access to work accommodations with increased employment retention and RTW among workers living with a chronic disease, while also demonstrating a negative correlation with long-term sick leave.^23^ In Canada, in accordance with human-rights informed legislation, employers are mandated to implement reasonable adjustments. Nonetheless, our findings highlighted concerns about job discrimination as barriers to disclosure, including fears of job loss and restricted access to promotions. Findings from a study conducted by our research team also revealed that concerns about organizational injustice predicted the duration of sick leave after kidney transplantation (K. Gaudet, MSc, M. Corbiere, PhD, S. Collette, MD, T. Chen, MD, H. Cardinal, MD, R. Sapir-Pichhadze, MD, Achille, M., PhD, unpublished data, December 2023).

Both before and after transplantation, workplace flexibility and workload reduction helped participants address the conflicting demands of work and illness-management on their time and energy. A previous qualitative study similarly emphacized the role of workplace flexibility on transplant recipients’ ability to manage their symptoms.^9^ The present findings also stress the importance of workers accessing flexibility in healthcare provision, such as access to night dialysis shifts and remote medical appointments. Importantly, workplace flexibility was not available for workers in technical and manual occupations, which underscores the significance of healthcare flexibility for these individuals.

Participants described that consulting their pre and posttransplantation nephrologist, hepatologist, or pulmonologist helped them assess their work ability and implement work accommodations. Previous studies remarked on physicians’ role in the latter.^14,16^ Significant differences have been found between healthcare professionals’ perceptions of transplant recipients’ work ability and recipients’ own perceptions.^24^ Our results build on this finding by suggesting that this discrepancy might be used at workers’ advantage if the patient-provider relationship is collaborative. More specifically, findings pointed to the existence of a climate of open dialogue and mutual influence, where workers felt that their points of view was valued and where they considered healthcare professionals’ advice.

While most participants talked about their work limitations in the workplace, many did not want to be perceived as sick. Other studies have suggested that workers pre and posttransplantation who rejected the sick role by focusing on their abilities rather than on their limitations felt more motivated and performed better at work.^16,25^ The present findings highlight that effective workplace communication, particularly in terms of requesting direct dialogue about task loads, helped workers distance themselves from the sick role, ultimately improving their work functioning. Participants posttransplantation also emphasized asking their co-workers to take precautions aimed at limiting their exposure to infections. Transplant-related anxiety, including fears about immunosuppression, has been shown to contribute to lower work functioning.^26^ The present results suggest that certain communication styles, such as being direct and systematic, may have a role in workers feeling safer in the workplace, which could facilitate their RTW by lowering anxiety about infection-induced organ rejection.

The second set of strategies involved a shifting prioritization of health versus work depending on workers’ stage in their transplantation trajectory. Before transplantation, participants withdrew from off-work activities and took time off when needed to manage ESOD-related fatigue and prevent increases in work disability. This is consistent with prior studies highlighting risks of health decline in workers with an ESOD who prioritized work over symptom management.^15,27^ The present findings indicated that workers awaiting transplantation were also preoccupied with losing access to surgery if they got injured or exhausted. Not going on sick leave earlier also negatively impacted some participants’ health and well-being. This highlights apparent limits to the use of self-management strategies as an ESOD progresses. After transplantation, like in previous studies,^8^ participants prioritized ensuring transplant longevity by proactively tending to their health. They also emphasized safeguarding time for enjoying the quality of life that it afforded them. The present findings adds to this research by suggesting that access to work accommodations, supporting long-term health and quality of life, is a non-negotiable RTW condition evoked by transplant recipients. In summary, findings highlighted an evoution from prioritizing labor participation at the expense of health and well-being pretransplantation to actively seeking a harmonious work-life balance that allowed for sustainable health promotion upon returning to work posttransplantation.

### Implications for Healthcare Providers

Healthcare managers may inquire into ways to integrate time-efficient care structures aimed at alleviating time drains for workers. Initiatives like prioritizing workers in choosing their in-center dialysis shifts, standardizing remote appointments, making blood work and other routine tests closer to workplaces or residences, along with reducing waiting times in healthcare facilities, could enhance efficiency.

Extensive literature has underscored the role of healthcare professionals in providing self-management support.^28^ The results from this study suggest that patients may find value in receiving education on fatigue and infection risk management, areas for which transplantation professionals are trained.^29^ Professionals could also play a role in helping patients identify their needs and advocate for themselves in the workplace. For instance, as illness-management tasks become burdensome before transplantation and during posttransplantation recovery, physicians and other healthcare professionals could prompt discussions about work limitations. Such discussions could include problem solving about barriers to implementing work accommodations, for example, by assisting claims to employers and insurance providers.

Before transplantation, workers sometimes put their health at risk by pushing their body beyond its limits. It could be helpful for healthcare professionals to promote open dialogue about the health impacts of work maintenance. Some of these health impacts might not fall into the category of occupational risk and thus might not be systematically discussed. In instances when workers struggle to accept their work limitations and are ambivalent about sick leave or disclosing their illness to managers, showing interest in their internal conflicts could be useful. Examples of such internal conflicts based on our findings include workers feeling like going on sick leave would equate to giving in to the sick role, or wanting to disclose their illness at work but fearing organizational injustice.

### Limitations and Suggestions for Future Research

This study's sample was comprised mostly of kidney transplant recipients. Some of its results are therefore centered around end-stage kidney disease experiences, such as dialysis. Individuals with end-stage liver, lung and heart diseases deal with higher levels of functional disability.^6^ Future studies could target these populations and seek to understand how disease-specific symptoms and limitations interact with workers’ support-seeking behaviors. The present study focused on workers’ perceptions. Future studies could aim to identify the strategies used by healthcare professionals, hospital administrators, and immediate supervisors when attempting to support workers’ sustained employment.

## Conclusion

Disclosure to immediate supervisors soon after the onset of ESOD gave participants early access to work flexibility, which in turn facilitated medical appointment attendance and fatigue management. Workload reduction further helped prevent risks of injury or exhaustion, yet some health impacts of staying at work persisted before transplantation. Support from healthcare professionals and flexibility in treatment and appointment schedules played a role in workers’ ability to manage their health while staying employed before and after solid organ transplantation. Future studies could describe the strategies used by other important stakeholders when providing support to workers.
